# Longitudinal ultrasound-based AI model predicts axillary lymph node response to neoadjuvant chemotherapy in breast cancer: a multicenter study

**DOI:** 10.1007/s00330-024-10786-5

**Published:** 2024-05-10

**Authors:** Ying Fu, Yu-Tao Lei, Yu-Hong Huang, Fang Mei, Song Wang, Kun Yan, Yi-Hua Wang, Yi-Han Ma, Li-Gang Cui

**Affiliations:** 1 Department of Ultrasound, Peking University Third Hospital, No. 49 North Garden Road, Haidian District Beijing, 100191 China; 2 Department of General Surgery, Peking University Third Hospital, No. 49 North Garden Road, Haidian District Beijing, 100191 China; 3 Department of Breast Cancer, Cancer Center, Guangdong Provincial People’s Hospital (Guangdong Academy of Medical Sciences), Southern Medical University, Guangzhou 510080 Guangdong, China; 4 Department of Pathology, Peking University Third Hospital, School of Basic Medical Sciences, Peking University Health Science Center, Beijing, 100191 China; 5 Department of Ultrasound, Peking University Cancer Hospital & Institute, Key Laboratory of Carcinogenesis and Translational Research (Ministry of Education/Beijing), No. 52 Fucheng Road, Haidian District Beijing, 100142 China; 6 Department of Ultrasound, North China University of Science and Technology Affiliated Hospital, 73 South Jianshe Road, Lubei District Tangshan, 066300 China

**Keywords:** Breast cancer, Ultrasonography, Axillary lymph node metastasis, Artificial intelligence, Neoadjuvant chemotherapy

## Abstract

**Objectives:**

Developing a deep learning radiomics model from longitudinal breast ultrasound and sonographer’s axillary ultrasound diagnosis for predicting axillary lymph node (ALN) response to neoadjuvant chemotherapy (NAC) in breast cancer.

**Methods:**

Breast cancer patients undergoing NAC followed by surgery were recruited from three centers between November 2016 and December 2022. We collected ultrasound images for extracting tumor-derived radiomics and deep learning features, selecting quantitative features through various methods. Two machine learning models based on random forest were developed using pre-NAC and post-NAC features. A support vector machine integrated these data into a fusion model, evaluated via the area under the curve (AUC), decision curve analysis, and calibration curves. We compared the fusion model’s performance against sonographer’s diagnosis from pre-NAC and post-NAC axillary ultrasonography, referencing histological outcomes from sentinel lymph node biopsy or axillary lymph node dissection.

**Results:**

In the validation cohort, the fusion model outperformed both pre-NAC (AUC: 0.899 vs. 0.786, *p* < 0.001) and post-NAC models (AUC: 0.899 vs. 0.853, *p* = 0.014), as well as the sonographer’s diagnosis of ALN status on pre-NAC and post-NAC axillary ultrasonography (AUC: 0.899 vs. 0.719, *p* < 0.001). Decision curve analysis revealed patient benefits from the fusion model across threshold probabilities from 0.02 to 0.98. The model also enhanced sonographer’s diagnostic ability, increasing accuracy from 71.9% to 79.2%.

**Conclusion:**

The deep learning radiomics model accurately predicted the ALN response to NAC in breast cancer. Furthermore, the model will assist sonographers to improve their diagnostic ability on ALN status before surgery.

**Clinical relevance statement:**

Our AI model based on pre- and post-neoadjuvant chemotherapy ultrasound can accurately predict axillary lymph node metastasis and assist sonographer’s axillary diagnosis.

**Key Points:**

- *Axillary lymph node metastasis status affects the choice of surgical treatment, and currently relies on subjective ultrasound*.
- *Our AI model outperformed sonographer’s visual diagnosis on axillary ultrasound*.
- *Our deep learning radiomics model can improve sonographers’ diagnosis and might assist in surgical decision-making*.

## Introduction

Neoadjuvant chemotherapy (NAC) is increasingly used for breast cancer with clinically positive axillary lymph nodes (ALN) [1, 2], necessitating accurate ALN response assessment for optimal post-NAC axillary surgical strategy [3]. While axillary lymph node dissection (ALND) remains the standard for clinical node-positive (cN+) breast cancer, NAC effectively eliminates ALN metastasis in 40–75% of cases [4]. Accurately predicting ALN response to NAC can markedly reduce unnecessary axillary surgeries and their associated risks like lymph node edema, and infection. Some patients may undergo axillary surgery despite without ALN metastasis [5].

Mammography, magnetic resonance imaging (MRI), and ultrasonography (US) are widely used to stage and monitor breast cancer during NAC treatment [6]. Radiomics is effective in cancer diagnosis, treatment evaluation, ALN metastasis detection, phenotype characterization, and prognosis prediction [7–12]. Deep learning offers automated, enhanced imaging feature analysis compared to traditional radiomics. In addition, transfer learning is explored for feature extraction in small medical datasets. Recent evidence suggests that deep learning radiomics (DLR) from preoperative US can predict early-stage breast cancer’s ALN status with high sensitivity and negative predictive value [13]. A study also found that a longitudinal MRI-based DLR model could predict the pathological complete response of breast cancer to NAC accurately, indicating that longitudinal medical images could capture more quantitative information during NAC [14]. Based on these findings, we hypothesize that a DLR model using pre-NAC and post-NAC US images can more effectively predict ALN response.

Few studies have trained and validated a multimodal DLR model that uses both pre-NAC and post-NAC ultrasound images to predict ALN response in breast cancer. Prior research has not compared artificial intelligence (AI) models with sonographers’ visual diagnosis on pre-NAC and post-NAC axillary ultrasound images. Our study focuses on comparing the DLR model’s predictive performance against sonographers, validating the model with independent external datasets, and assessing the AI model’s potential to improve sonographers’ diagnostic ability in axillary diagnosis on ultrasound images.

## Materials and methods

### Patients

**Fig. 1:**
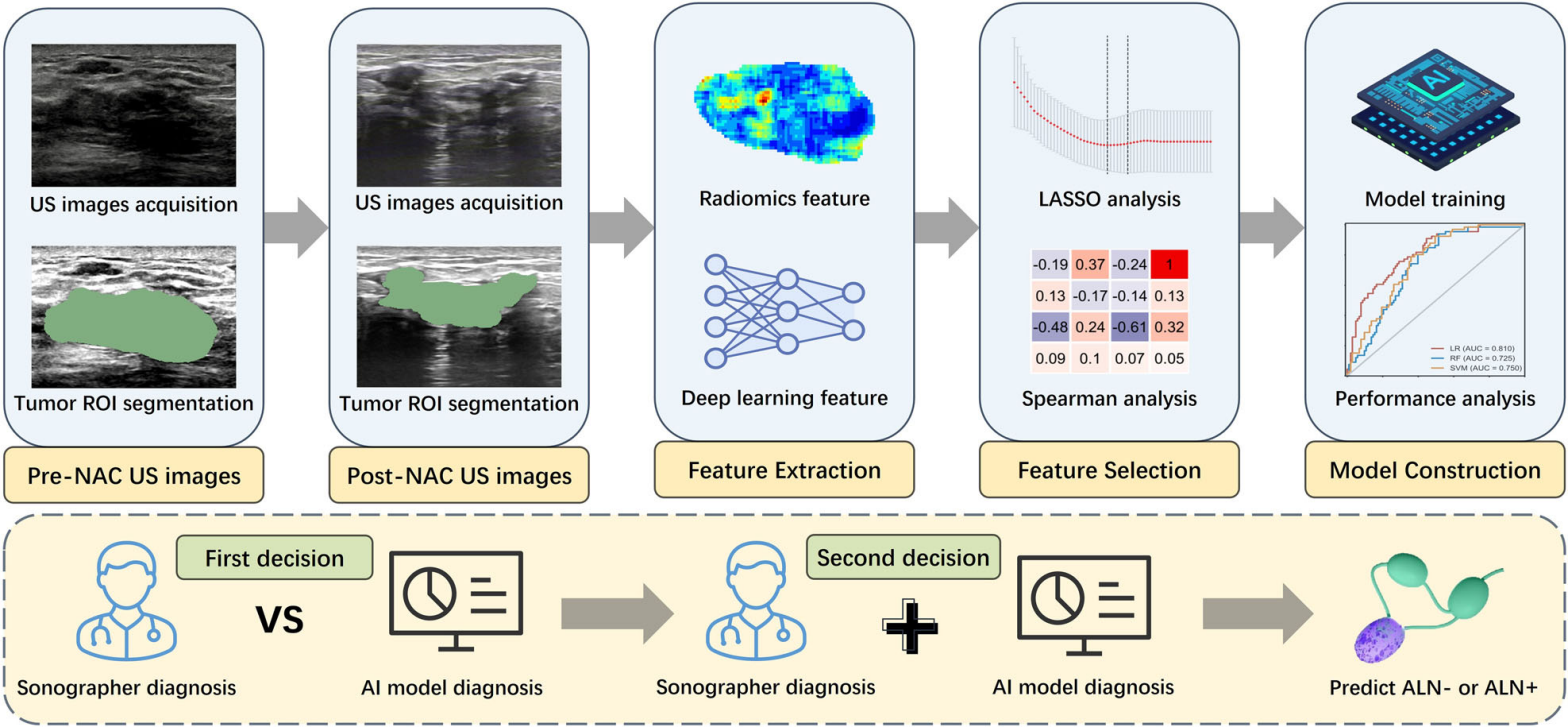
The design of the workflow for this study. The construction of the deep learning radiomics model involves the following steps: Acquisition of original images, manual segmentation, feature extraction, feature selection, the model construction. A sonographers made the first decision of axillary lymph node status using the pre-NAC and post-NAC axillary ultrasound. After a month interval, a second decision was provided for the same images with the assistance of an artificial intelligence model. The pathological results of axillary lymph nodes were regarded as the golden standard. NAC, neoadjuvant chemotherapy; US, ultrasound; AI, artificial intelligence; ROI, region of interest; ALN, axillary lymph node; LASSO, least absolute shrinkage and selection operator

The study received ethical approval from the Ethics Committees of the Peking University Third Hospital, Guangdong Provincial People’s Hospital, and Peking University Cancer Hospital. Due to the retrospective nature of the study, patient informed consent was waived. From November 2016 to December 2022, 669 patients from three hospitals, who underwent NAC followed by surgery, were enrolled. The inclusion criteria were: (i) cN+ breast cancer treated with standard NAC; (ii) complete pre-NAC and post-NAC ultrasound scans; (iii) ALN staging via sentinel lymph node biopsy (SLNB) or ALND; and (iv) complete baseline data. The exclusion criteria were: (i) prior breast cancer treatment (*n* = 43), (ii) other malignancies or distant metastasis (*n* = 28), (iii) bilateral breast cancer (*n* = 18), (iv) inadequate or poor-quality US images (*n* = 39), and (v) missing clinicopathological data (*n* = 44). Patients from hospitals I and II comprised the training cohort (*n* = 216), whereas patients from hospitals III comprised the independent validation cohort (*n* = 281). Figure 1 shows the study workflow.

### NAC regimen and histological assessment

All patients underwent 6 or 8 cycles of NAC, using either taxane alone or in combination with anthracycline, with human epidermal growth factor receptor-2 (HER2) positive patients also receiving anti-HER2 therapy. Surgery followed 2–3 weeks after NAC. ALN response to NAC was assessed histologically via SLNB or ALND, defining ALN metastasis as invasive tumor presence in any lymph node. Immunohistochemistry (IHC) determined HER2, hormone receptor (HR), and Ki-67 status: estrogen receptor (ER) and progesterone receptor (PR) were positive if staining cells > 1%, while Ki-67 expression was defined as high or low with a 20% cutoff [15]. HER2 status was based on IHC scores (0 or 1+ as negative, 3+ as positive) or fluorescence in situ hybridization for IHC 2+ cases [16]. Baseline data, including age, menstrual status, clinical T and N stages, were recorded. Breast cancer was classified into HR+/HER2-, HER2+, and TNBC based on molecular receptor expression.

### Ultrasound examination

All patients underwent pre- and post-NAC ultrasound examinations conducted two weeks before and after NAC treatment. Ultrasound images were obtained using Esaote (MyLab Twice), Siemens (S3000), or Philips (EPIQ5) ultrasound scanners equipped with 7- to 15-MHz linear transducer (see Supporting Material-II, Ultrasound Examinations). Two sonographers performed ultrasound examinations at hospital I, one at hospital II, and two at hospitals III. Each sonographer had more than eight years of experience in breast ultrasound imaging. Before NAC, patients underwent breast ultrasound scans and core-needle biopsy, selecting the ultrasound images containing the largest diameter of mass for further analysis. A total of 2585 ultrasound images from 497 patients were collected and analyzed, encompassing both pre-NAC and post-NAC images.

### Tumor segmentation and image preprocessing

Pre-NAC and post-NAC US images were imported into 3D Slicer software (version 4.10.1) for manual tumor delineation. Two experienced sonographers (6 and 8 years in breast cancer ultrasound), blinded to histological results, segmented the tumor regions of interest (ROI), encompassing the entire tumor but excluding blood vessels, adipose tissue, and normal breast tissue. Each ultrasound image had its tumor ROI delineated. For radiomics process, US images were preprocessed to a uniform voxel size of 1 × 1 mm. For the deep learning process, US images covering the entire tumor area were resized to 448 × 448 pixels and grayscale normalized to 0–1000 for uniform feature extraction.

### Feature extraction and selection

Feature extraction and selection were conducted on pre-NAC and post-NAC ultrasound images using pyradiomics software (version 3.3.0), extracting 2446 radiomics features (1223 from each pre-NAC and post-NAC; see Supporting Material-I. Feature Extraction). These included shape-based, first-order statistical, texture-based, and wavelet-derived features. Combat harmonization minimized bias from different scanners across hospitals. For deep learning, all resized images were input into the deep convolutional neural network VGG16, which has been pretrained on a large-scale ImageNet database (https://www.image-net.org/). Then we extracted transfer features from the fully connected layers (see Supporting Material-III. Basic Principles of Deep Learning and Neural Network). This yielded 1223 pre-NAC and 1223 post-NAC radiomic features, and 2048 deep learning features each for pre-NAC and post-NAC.

Feature values were standardized using *z*-score normalization. In the training cohort, feature selection involved the Mann–Whitney *U* test to identify features significantly associated with ALN response to NAC (*p* < 0.05). The Least Absolute Shrinkage and Selection Operator (LASSO) was used to eliminate features with zero coefficients. To reduce feature correlation, Spearman analysis removed one feature from highly correlated pairs (correlation coefficient > 0.8), based on their diagnostic performance.

### Model construction and Integration

To optimize the DLR model for ALN response prediction after NAC, we adjusted their hyperparameters. This included hyperparameter tuning to enhance model performance and early stopping to prevent model overfitting, ensuring model generalizability. We used 30% of the training cohort to assess the VGG16 model’s performance, measured by the area under the curve (AUC), and stopped training if the performance did not increase over ten consecutive calculation cycles. To further refine the model, significant conventional ultrasound features such as tumor size, echo type, and blood flow signal, were integrated into the fully connected layer, increasing neuron count. Two predictive models (pre-NAC and post-NAC) were built using a random forest algorithm, generating two DLR signatures. A support vector machine (SVM) model then combined pre-NAC and post-NAC radiomics and deep learning features. The integration of these temporally distinct features enables a more comprehensive analysis, enhancing the machine learning model’s predictive power. The SVM model was designed to accurately predict the ALN metastasis in breast cancer patients following NAC.

### Comparison with sonographer and AI-assisted diagnosis

We evaluated model performance by comparing each machine learning model’s AUC with sonographer’s diagnosis on axillary ultrasound and explored if the fusion model enhanced sonographer’s diagnostic ability. Two sonographers, with 6 and 8 years of experience, independently assessed ALN status on pre-NAC and post-NAC ultrasound images, blinded to pathological results. Based on previous studies, the presence of any of the following criteria indicates metastatic ALN on US: (i) loss of the fatty hilum, (ii) round shape, or (iii) eccentric cortical thickening (> 3 mm) [17, 18]. After a month, the same sonographers re-assessed the US images with AI model assistance, initially obtaining the AI prediction before making their final diagnosis. We compared the sonographer’s initial diagnosis with the AI-assisted diagnosis to determine whether the AI model would serve as a useful tool for enhancing the sonographer’s diagnostic ability.

### Statistical analysis

Statistical analysis was conducted using SPSS software (version 25.0). Group differences were assessed using the student’s *t*-test or Mann-Whitney *U*-test for continuous variables and the chi-square test or Fisher’s exact test for categorical variables. The performances of the models were evaluated using the AUC, and the DeLong test was used to compare the performances of the different models. Decision curve analysis (DCA) to evaluate the clinical utility of the models [19]. Model performance was assessed based on accuracy (ACC), specificity (SPE), sensitivity (SEN), positive predictive value (PPV), and negative predictive value (NPV), seeing Supporting Material-IV. Statistical Metrics. Statistical significance was set at *p* < 0.05.

## Results

### Baseline characteristics of patients

In this study, 497 patients were included, with an average age of 51.47 years. Of these patients, 210 were ALN+ and 287 were ALN- after NAC. The ALN+ rates were 51.39% in the training cohort and 35.23% in the validation cohort. Significant differences in molecular subtype, primary tumor response and clinical N stage were observed between ALN+ and ALN- groups (all the *p* < 0.05), while other baseline characteristics showed no significant variance in both training and validation cohorts. Table 1 details the baseline characteristics of the patients.

**Table 1:** Clinicopathologic characteristics of patients in the ALN+ and ALN- groups

Characteristics	Training cohort		*p* value	Validation cohort		*p* value
	ALN− (*n* = 105)	ALN+ (*n* = 111)		ALN− (*n* = 182)	ALN+ (*n* = 99)	
Age, mean ± sd	51.16 ± 11.06	51.88 ± 11.62	0.641	49.36 ± 9.93	47.99 ± 9.60	0.262
Menstrual status, *n* (%)			0.861			0.794
yes	47 (44.8%)	51 (45.9%)		82 (45.1%)	43 (43.4%)	
no	58 (53.2%)	60 (54.1%)		100 (54.9%)	56 (56.6%)	
Molecular subtype, *n* (%)			< 0.001			< 0.001
TNBC	16 (15.2%)	16 (14.4%)		36 (19.8%)	19 (19.2%)	
HR+/HER2−	33 (31.4%)	68 (61.3%)		41 (22.5%)	58 (58.6%)	
HER2+	56 (53.4%)	27 (24.3%)		105 (57.7%)	22 (22.2%)	
HR receptor, *n* (%)			0.031			< 0.001
HR−	44 (41.9%)	31 (27.9%)		88 (48.4%)	26 (26.3%)	
HR+	61 (58.1%)	80 (72.1%)		94 (51.6%)	73 (73.7%)	
HER2 receptor, *n* (%)			< 0.001			< 0.001
HER2−	49 (46.7%)	84 (75.7%)		77 (42.3%)	77 (77.8%)	
HER2+	56 (53.3%)	27 (24.3%)		105 (57.7%)	22 (22.2%)	
Tumor response, *n* (%)			< 0.001			< 0.001
pCR	48 (45.7%)	18 (16.2%)		77 (42.3%)	11 (11.1%)	
non-pCR	57 (54.3%)	93 (83.8%)		105 (57.7%)	88 (88.9%)	
Clinical T stage, *n* (%)			0.058			0.056
cT1	21 (20.0%)	15 (13.5%)		23 (12.6%)	6 (6.1%)	
cT2	70 (66.7%)	70 (63.1%)		117 (64.3%)	58 (58.6%)	
cT3	9 (8.6%)	23 (20.7%)		20 (11.0%)	13 (13.1%)	
cT4	5 (4.7%)	3 (2.7%)		22 (12.1%)	22 (22.2%)	
Clinical N stage, *n* (%)			< 0.001			0.002
cN1	88 (83.8%)	79 (71.2%)		147 (80.8%)	69 (69.7%)	
cN2	13 (12.4%)	27 (24.3%)		25 (13.7%)	16 (16.2%)	
cN3	4 (3.8%)	5 (4.5%)		10 (5.5%)	14 (14.1%)	

Golden standard for the definition of a lymph node metastasis was histology following to SLNB or ALND after NAC

*ALN+* Axially lymph node metastasis, *ALN−* Axially lymph node without metastasis, *HER2+* human epidermal growth factor receptor-2, *TNBC* triple negative breast cancer, *HR* hormone receptor, *pCR* pathological complete response, *NAC* neoadjuvant chemotherapy, *SLNB* sentinel lymph node biopsy, *ALND* axillary lymph node dissection

### Feature selection and model construction

In the training cohort, 1362 radiomic features (463 pre-NAC, 899 post-NAC) and 2908 deep learning features (1357 pre-NAC, 1551 post-NAC) from ultrasound images were significantly associated with ALN metastasis after NAC (Mann–Whitney *U* test, *p* < 0.05). After LASSO selection, seven pre-NAC and nine post-NAC features were selected. The detailed LASSO selection mean-square error change curve and coefficient change lines are shown in Fig. S1. From highly correlated pairs (Spearman correlation coefficient > 0.8), the feature with higher diagnostic performance was retained, resulting in six pre-NAC and eight post-NAC features for model construction (see Table 2). Two random forest models (pre-NAC and post-NAC) were developed, with their output signatures integrated into a SVM model.

**Table 2:** The details of selected radiomics and deep learning features for model construction, including ICC value and LASSO coefficient of features

Original image	Feature type	Feature name	ICC value	LASSO coefficient
Pre-NAC image	Rad_ori_firstorder	Maximum	0.948	0.0212
	Rad_ori_GLRLM	LongRunLowGrayLevelEmphasis	0.904	−0.01085
	Rad_wavalet_GLSZM	GrayLevelVariance	0.913	−0.0224
	Deep learning	VGG16_98	0.907	0.00325
	Deep learning	VGG16_972	0.974	0.0208
	Deep learning	VGG16_1679	0.876	−0.00916
Post-NAC image	Rad_ori_firstorder	Entropy	0.961	−0.0367
	Rad_wavalet_GLCM	IDM	0.977	0.0211
	Rad_wavalet_GLDM	LowGrayLevelEmphasis	0.901	0.0127
	Deep learning	VGG16_230	0.913	0.00293
	Deep learning	VGG16_384	0.905	−0.0450
	Deep learning	VGG16_719	0.955	0.00751
	Deep learning	VGG16_1625	0.924	0.0422
	Deep learning	VGG16_1832	0.932	−0.0493

*ICC* intraclass correlation coefficient, *LASSO* least absolute shrinkage and selection operator, *NAC* neoadjuvant chemotherapy, *Rad* radiomics, *GLRLM* gray level run length matrix, *GLSZM* gray level size zone matrix, *GLCM* gray level co-occurrence matrix, *GLDM* gray level dependence matrix

**Fig. 2:**
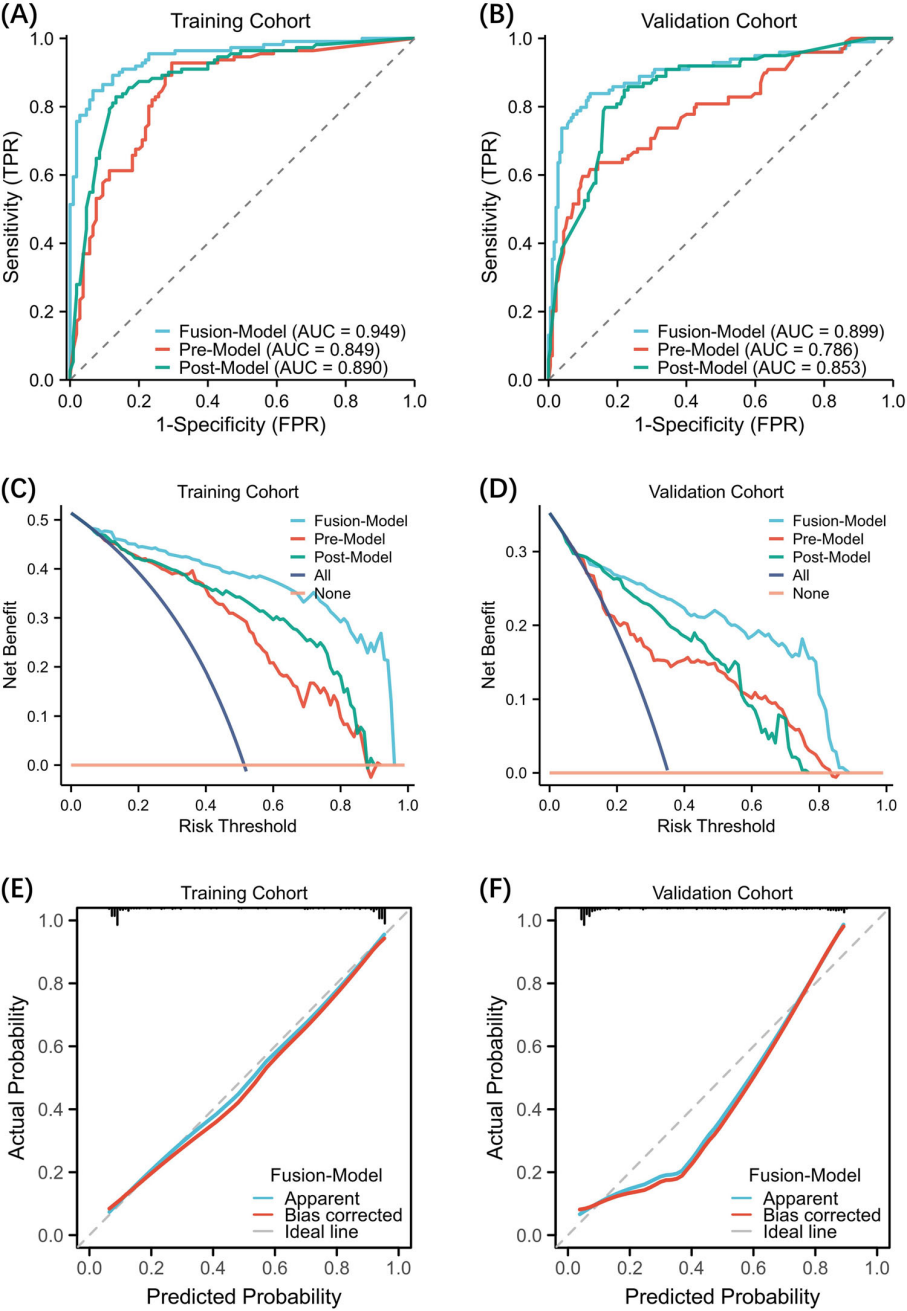
Comparison of ROC curves, Decision curve analysis of the three models, and the calibration curves of the fusion model. ROC curves show the performance of the fusion model, pre-NAC model, and post-NAC model for predicting ALN metastasis in the training (**A**) and validation cohorts (**B**). Decision curve analysis (DCA) for three models was showed in the training (**C**) and validation cohorts (**D**), the y-axis indicates the net benefit; x-axis indicates threshold probability. Calibration curves of the fusion model in the training (**E**) and validation (**F**) cohorts are presented. AUC, area under the curve; FPR, false positive rate; TPR, true positive rate

Figure 2A, B show the ROC curves of the three machine learning models, with the fusion model achieving the highest AUCs of 0.949 in the training cohort and 0.899 in the validation cohort. It outperformed both the pre-NAC (AUC = 0.786, *p* < 0.05) and post-NAC (AUC = 0.853, *p* < 0.05) models in the validation cohort. The decision curve analysis demonstrated that the combined model had satisfactory net clinical benefits in both the training and validation cohorts (Fig. 2C, D). The calibration plots also demonstrated excellent agreement between the actual and predicted ALN status in both cohorts of the fusion model (Fig. 2E, F).

### Comparison of sonographer and radiomics model

Our study compared the sonographers’ first diagnosis on axillary ultrasound with three machine-learning models based on pre-NAC and post-NAC breast ultrasound features. The models’ performance metrics, including AUC, ACC, SEN, SPE, PPV, and NPV, are detailed in Table 3. The fusion model outperformed the sonographer in the training cohort with an accuracy of 88.89%, sensitivity of 84.68%, and specificity of 93.33%, and in the validation cohort with an accuracy of 85.77%, sensitivity of 83.84%, and NPV of 86.81%. Despite its wide clinical application, axillary ultrasound showed the lowest AUCs (0.753 in training cohort, 0.719 in validation cohort). The three AI models (AUCs: 0.899, 0.786, and 0.853, respectively) surpassed the sonographer’s first diagnosis (AUC: 0.719) in the validation cohort. The sonographer identified ALN+ patients with sensitivities of 63.06% (training cohort) and 51.52% (validation cohort), while AI models achieved higher sensitivities (82.88–92.79% in training cohort, 73.73–83.84% in validation cohort). For identifying ALN- patients, the sonographer’s specificity was comparable to the AI models, with the pre-NAC model showing the lowest specificity (70.48% in training cohort, 64.84% in validation cohort).

**Table 3:** The performance of different models and sonographer in training and validation cohorts

Cohort	Approach	AUC (95% CI)	ACC (%)	SEN (%)	SPE (%)	PPV (%)	NPV (%)	Delong test (*p*)
Training (*n* = 216)	Fusion model	0.949 (0.921, 0.977)	88.89	84.68	93.33	93.07	85.22	Reference
	Pre-NAC model	0.849 (0.797, 0.902)	81.94	92.79	70.48	76.87	90.24	< 0.001
	Post-NAC model	0.890 (0.845, 0.936)	84.72	82.88	86.67	86.79	82.73	0.091
	Sonographer	0.753 (0.698, 0.809)	75.00	63.06	87.62	84.34	69.17	< 0.001
	Sonographer + AI	0.813 (0.764, 0.863)	81.02	70.27	92.38	90.70	74.62	< 0.001
Validation (*n* = 281)	Fusion model	0.899 (0.855, 0.943)	85.77	83.84	86.81	77.57	90.80	Reference
	Pre-NAC model	0.786 (0.728, 0.844)	67.97	73.74	64.84	53.28	81.94	< 0.001
	Post-NAC model	0.853 (0.806, 0.901)	80.07	80.81	79.67	68.38	88.41	0.014
	Sonographer	0.719 (0.663, 0.774)	74.02	51.52	86.26	67.11	76.59	< 0.001
	Sonographer + AI	0.792 (0.740, 0.844)	81.49	62.63	91.76	80.52	81.86	< 0.001

*AUC* area under the curve, *ACC* accuracy, *SEN* sensitivity, *SPE* specificity, *PPV* positive predictive value, *NPV* negative predictive value

### AI assist in sonographer’s diagnosis on ALN status

**Fig. 3:**
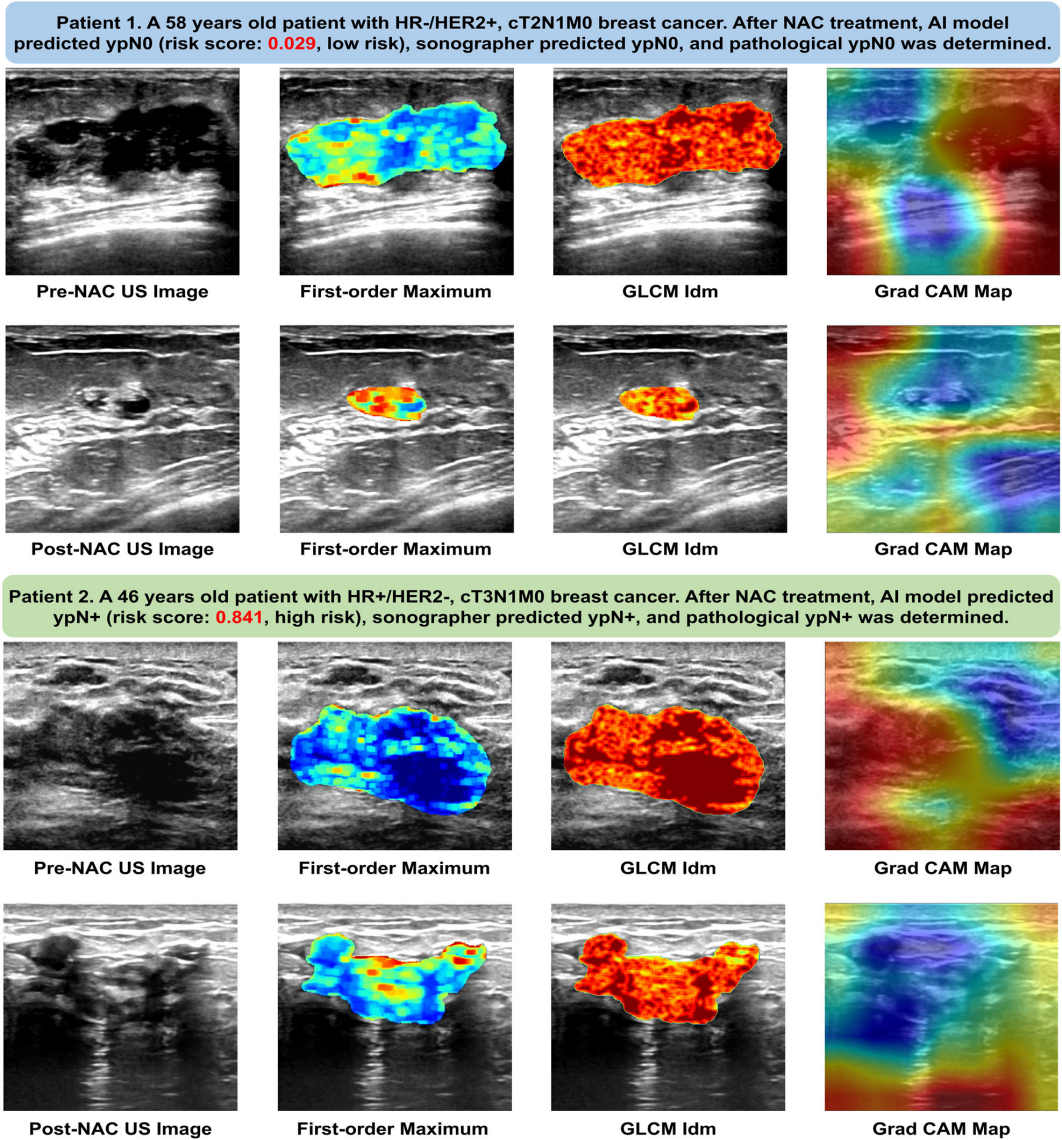
This Figure illustrates pre- and post-neoadjuvant chemotherapy (NAC) ultrasound images from two patients: one showing a complete axillary lymph node (ALN) response and the other with residual ALN metastasis after NAC. The second and third columns correspond to the radiomics heatmap (Firstorder_Maximum and GLCM_IDM) generated from the radiomics pipeline, while the fourth column depicts the Grad CAM Map heatmap from the deep learning pipeline. These heatmaps visually represent areas of interest identified by each model in assessing ALN status. For the Firstorder_Maximum heatmap, the larger the dark blue prompt value is, the more disordered the intensity value is in this region. For the GLCM_IDM heatmap, the larger the dark red prompt value is, the more disordered the texture is in this region. For the Grad CAM heatmap, the larger the red prompt value is, the more contribution feature value is in this region, also indicating the deep learning model pays more attention to the red region on breast cancer ultrasound image. NAC, neoadjuvant chemotherapy; GLCM, Gray level co-occurrence matrix inverse difference moment; Grad CAM, gradient-weighted class activation mapping; US, ultrasound; HR, hormone receptor; HER2, human epidermal growth factor antibody 2; AI, artificial intelligence

With the assistance of the fusion AI model, the sonographer performed a second reading of the US image. As seen in Table 3, the sonographer’s diagnostic ability improved when assisted by the AI model, most prominently in sensitivity, which increased from 63.06% to 70.27% in the training cohort and from 51.52% to 62.63% in the validation cohort. Moreover, the AUCs of the sonographer’s second diagnosis were considerably greater than that of the initial diagnosis (*p* < 0.05 in both the training and validation cohorts), indicating that the fusion AI model effectively improved the sonographer’s diagnostic ability. Figure 3 illustrates the ROI delineation and heatmap on the US images of two representative patients (ALN+ and ALN−).

## Discussion

ALN status is crucial for guiding surgical treatment in clinical practice, as ALN metastasis typically indicates a worse prognosis and a higher recurrence risk [6]. SLNB or ALND is routinely performed to assess the axillary lymph node status. In our study, 42.25% of the patients had no ALN metastasis after NAC but underwent invasive axillary surgery, leading to huge costs and unnecessary complications. Previous studies have confirmed that MRI-based radiomics features from primary tumors could accurately predict the ALN status with an AUC of 0.790–0.862, but only focused on imaging-derived radiomics [15, 20–22]. A previous study showed the feasibility of predicting the ALN status using a mammography-based radiomics model with an AUC of 0.809 (95% CI, 0.794–0.833) [23]. Our study involved developing a multimodality AI model using pre- and post-NAC US images, allowing for a more comprehensive use of US images to predict ALN status. The DeLong test revealed the fusion model’s reliability in noninvasively identifying the ALN status after NAC, sparing unnecessary surgery and complications.

Axillary ultrasound is commonly used to evaluate the ALN status during NAC in patients with breast cancer. In our study, the fusion model demonstrated superior diagnostic performance, with an AUC of 0.899 in the validation cohort, significantly outperforming the sonographer’s diagnosis on axillary US, with an AUC of 0.719. Alvarez reported that axillary ultrasound’s sensitivity and specificity for breast cancer ranged from 48.8% to 87.1% and 55.6% to 97.3%, respectively, consistent with our findings [24]. However, axillary ultrasound diagnosis is usually influenced by the operator experience, and difficulty in detecting very small metastasis in the ALN region. Thus, despite its widespread clinical use, a sonographer’s ultrasound diagnosis should not be the only imaging approach for assessing ALN status after NAC. In addition, we found that the sonographer’s diagnosis on the axillary US showed high specificity for ALN diagnosis after NAC but low sensitivity. Our results are consistent with previous studies showing that the sensitivity of MRI was 61.4–70%, indicating that axillary US performed similarly to MRI [25, 26]. Moreover, sonographers rely on subjective judgments of ALN morphology, whereas respiratory and cardiac motion artifacts may affect their diagnosis on MRI. Conventional US images are also more robust.

In our study, the sonographers’ first diagnosis relied only on their personal perspective or personal diagnosis, whereas the second diagnosis referenced the prediction results of the fusion model. Some breast cancer heterogeneity might relate to ALN metastasis, but cannot be visually observed by sonographers. The results showed that the diagnostic ability was significantly enhanced in the second diagnosis, indicating that the AI model can capture and integrate potential breast cancer heterogeneity overlooked by sonographers when assessing ALN status. When the AI model’s risk score significantly deviates from the sonographer’s first diagnosis, the sonographer would pay more attention to the lymph nodes, which initially were indeterminate and were not classified as metastatic in the first reading. Sonographers re-evaluated and made an upgrading ALN diagnosis in the second reading with AI assistance.

The fusion model’s superiority for higher threshold probabilities above 15% suggests its utility in identifying patients who could benefit from ALND, thereby minimizing unnecessary surgical interventions. However, the ideal threshold for ALND recommendation should balance the risks of unwarranted surgery against undertreatment risks, warranting further validation in future studies tailored to patient conditions and clinical practices. In addition, the model’s high negative predictive value (NPV) of 90.8% in the validation cohort suggests its effectiveness in accurately identifying patients who may not need ALND, potentially averting related surgical complications. Nonetheless, ALND omission decisions should consider the AI model’s predictions in conjunction with other factors, including patient personalized condition, molecular subtype, and lymph node size.

Our study had some limitations. First, primary tumor segmentation was performed manually, which is time-consuming. In future, we plan to explore the performance of an automatic segmentation model. Second, selection bias was unavoidable due to the retrospective nature of the study. Larger sample sizes and evidence from more multicenter studies are required to test the predictive efficiency and assistive ability of the AI model. Third, we collected US images from various acquisition protocols, potentially affecting the imaging analysis. Thus, a harmonization process was employed to minimize heterogeneity. Finally, the relatively limited number of sonographers who participated in this study may not accurately represent an average sonographer’s ability. Future studies should involve more sonographers in diagnosing ALN status to evaluate the model’s assist efficacy more comprehensively.

## Conclusion

We developed a fusion AI model that integrates pre- and post-NAC US images, providing superior prediction of ALN metastasis after NAC in breast cancer compared with the single-modality model or sonographer diagnosis. This AI model can serve as an effective tool to assist sonographers in improving their diagnostic abilities.

## Supplementary information

Supplementary Materials

## Abbreviations

AI: Artificial intelligence
ACC: Accuracy
ALN: Axillary lymph node
ALND: Axillary lymph node dissection
AUC: Area under the curve
cN+: Clinical node-positive
DLR: Deep learning radiomics
HER2: Human epidermal growth factor receptor-2
HR: Hormone receptor
IHC: Immunohistochemical
LASSO: Least absolute shrinkage and selection operator
MRI: Magnetic resonance imaging
NAC: Neoadjuvant chemotherapy
NPV: Negative predictive value
PPV: Positive predictive value
ROC: Receiver operating characteristic
ROI: Regions of interest
SEN: Sensitivity
SLNB: Sentinel lymph node biopsy
SPE: Specificity
SVM: Support vector machine
US: Ultrasonography
