# Supplementary material for: Longitudinal ultrasound-based AI model predicts axillary lymph node response to neoadjuvant chemotherapy in breast cancer: a multicenter study
Source: Eur Radiol. 2024 May 10;34(11):7080–9. doi: 10.1007/s00330-024-10786-5 (PMC11519196; doi:10.1007/s00330-024-10786-5)

Supplementary Materials

(I) Supporting Material: Feature Extraction

Before features extraction, the voxel size of each sequence was resampled to 1 × 1 × 1 mm3 and the bin width of gray-level histogram was fixed as 25. After z-score normalization of image pixel intensities, a total of 1408 quantitative imaging features including 13 shape based features, 18 first order statistical features, 75 textural features from original images and 1302 derived features (744 features of Gabor-bank wavelet filtered images and 558 features of Law’s filtered images), were extracted respectively for T2 images, ADC maps and T1-DCE images using corresponding ROIs.

(1) Shape based features

In this group of features, we included descriptors of the three-dimensional shape and size of the tumor region. Let in the following definitions V denote the volume and A the surface area of the volume of interest. We determined the following shape and size based features:

1. **Compactness 1**= $\frac{V}{\sqrt{\pi} A^{\frac{2}{3}}}$
2. **Compactness 2**= $36\pi \frac{V^2}{A^3}$
3. **Maximum 3d diameter**: The maximum three-dimensional tumor diameter is measured as the largest pairwise Euclidean distance, between voxels on the surface of the tumor volume.
4. **Spherical disproportion** = $\frac{A}{4\pi R^2}$
5. **Sphericity** = $\frac{\pi^{\frac{1}{3}}(6V)^{\frac{2}{3}}}{A}$
6. **Surface area**: The surface area is calculated by triangulation (i.e. dividing the surface into connected triangles) and is defined as:

   $$A = \sum_{i=1}^{N} \frac{1}{2} \left| a_i b_i \times a_i c_i \right|$$

   Where N is the total number of triangles covering the surface and a, b and c are edge vectors of the triangles.

7. **Surface to volume ratio** = $\frac{A}{V}$

8. **Volume**: The volume (V) of the tumor is determined by counting the number of pixels in the tumor region and multiplying this value by the voxel size.

(2) First order statistical features

The following 17 statistical features were extracted.

Let **X** be the three dimensional image matrix with *N* voxels of the ROI and P be the first order histogram distribution with N_g discrete intensity levels.

1. **IntensityMax:** The maximum intensity value of **X.**
2. **IntensityMin:** The minimum intensity value of **X**.
3. **Median:** The median intensity value of **X**.
4. **IntensityStd:**

$$IntensityStd = \left(\frac{1}{L*W*H-1}\sum_{i=1}^{L}\sum_{j=1}^{W}\sum_{k=1}^{H}(X(\mathrm{i,j,k}) - IntensityAve)^2\right)^{1/2}$$

5. **Mean:**

$$\frac{1}{N}\sum_{i}^{N} X(i)$$

6. **Variance:**

$$\sqrt{\sum_{j=1}^{N_g} P(\mathrm{j}) * (\mathrm{j} - \sum_{i=1}^{N_g} P(\mathrm{i}) * \mathrm{i})^2}$$

7. **Skewness:**

$$\sqrt{\sum_{j=1}^{N_g} P(\mathrm{j}) * (\mathrm{j} - \sum_{i=1}^{N_g} P(\mathrm{i}) * \mathrm{i})^3}$$

8. **Kurtosis:**

$$\sqrt{\sum_{j=1}^{N_g} P(\mathrm{j}) * (\mathrm{j} - \sum_{i=1}^{N_g} P(\mathrm{i}) * \mathrm{i})^4}$$

9. **Range:**

   The range of intensity values of **X**.

10. **Mean absolute deviation:**

The mean of the absolute deviations of all voxel intensities around the mean intensity value

11. Energy:

$$\sum_{i}^{N} X(i)^2$$

12. Entropy:

$$\sum_{i=1}^{N_g} P(i)\log_2 P(i)$$

13. Entropy_p:

$$\sum_{i=1}^{N_g} \frac{P(i)}{N}\log_2 \frac{P(i)}{N}$$

14. Root mean square:

$$\sqrt{\frac{\sum_{i}^{N} X(i)^2}{N}}$$

15. Uniformity:

$$\sum_{i=1}^{N_g} P(i)^2$$

16. **Uniformity_p:**

$$\sum_{i=1}^{N_g} \left(\frac{P(i)}{N}\right)^2$$

17. Mass:

The sum intensity value of X.

(3) Textural features

Second order statistic texture features, and higher order statistic texture features were extracted. Forty-four second order statistic texture features could be calculated from the Gray Level Co-occurrence Matrix (GLCM). Forty-six high order statistic texture features were calculated from the Gray Level Size Zone Matrix (GLSZM), Gray Level Run Length Matrix (GLRLM), and Neighborhood Gray Tone Difference Matrix (NGTDM). All of the GLCM, GLSZM, GLRLM, and NGTDM based texture feature were calculated using a 2D analysis and then

averaged for all slices within the three-dimensional tumor volume.

Gray-Level Co-Occurrence Matrix based features (GLCM)

GLCM based features were second-order statistical texture features, which are defined as a matrix *M* (*i, j; δ, θ*) to indicate the relative frequency with intensity values of pixels (*i* and *j*) at the distance of *δ* in direction *θ*.

Let:

M(*i, j*) be the co-occurrence matrix for an arbitrary *δ* and *θ*, set *δ=1 and θ=0 and 45*

N_g be the number of discrete intensity levels in the images, set as 25,

μ be the mean of *M*(*i, j*),

$m_x(\mathrm{i}) = \sum_{j=1}^{N_g} M(\mathrm{i,j})$ be the marginal row probabilities,

$m_y(\mathrm{i}) = \sum_{i=1}^{N_g} M(\mathrm{i,j})$ be the marginal column probabilities, and u_y ,μ_x, be the mean of m_x .and m_y

$$HX = -\sum_{i=1}^{N_g} m_x(i) \log(m_x(i),$$

$$HY = -\sum_{i=1}^{N_g} m_y(i) \log(m_y(i),$$

$$HXY = -\sum_{i=1}^{N_g} \sum_{j=1}^{N_g} m(i,j) \log(m(i,j)),$$

$$HXY1 = -\sum_{i=1}^{N_g} \sum_{j=1}^{N_g} m(i,j) \log(m_x(i) m_y(j)).$$

$$HXY2 = -\sum_{i=1}^{N_g} \sum_{j=1}^{N_g} m_x(i) m_y(j) \log(m_x(i) m_y(j)).$$

1. **Energy:**

$$\sum_{i=1}^{N_g} \sum_{j=1}^{N_g} [\mathrm{M(i,j)}]^2$$

2. **Contrast:**

$$\sum_{i=1}^{N_g} \sum_{j=1}^{N_g} (\mathrm{i-j})^2 * M(\mathrm{i,j})$$

3. **Entropy:**

$$\sum_{i=1}^{N_g}\sum_{j=1}^{N_g} M(\mathrm{i},\mathrm{j}) * \log_2 M(\mathrm{i},\mathrm{j})$$

4. **Homogeneity 1:**

$$\sum_{i=1}^{N_g}\sum_{j=1}^{N_g} \frac{M(\mathrm{i},\mathrm{j})}{1+|\mathrm{i}-\mathrm{j}|}$$

5. **Homogeneity 2:**

$$\sum_{i=1}^{N_g}\sum_{j=1}^{N_g} \frac{M(\mathrm{i},\mathrm{j})}{1+|\mathrm{i}-\mathrm{j}|^2}$$

6. **Correlation:**

$$\frac{\sum_{i=1}^{N_g}\sum_{j=1}^{N_g} ijM(\mathrm{i},\mathrm{j}) - \mu_i(\mathrm{i})\mu_j(\mathrm{j})}{\sigma_x(\mathrm{i})\sigma_y(\mathrm{j})}$$

7. **Variance:**

$$\sum_{i=1}^{N_g}\sum_{j=1}^{N_g} (\mathrm{i}-\mu)^2 M(\mathrm{i},\mathrm{j})$$

8. **Sum Average:**

$$\sum_{i=2}^{2N_g} iM_{x+y}(\mathrm{i})$$

9. **Sum Entropy:**

$$-\sum_{i=2}^{2N_g} M_{x+y}(\mathrm{i})\log_2\left[M_{x+y}(\mathrm{i})\right]$$

10. **Dissimilarity:**

$$\sum_{i=1}^{N_g}\sum_{j=1}^{N_g} |\mathrm{i}-\mathrm{j}|\,\mathrm{M(i,j)}$$

11. **Inverse Difference Moment:**

$$\sum_{i=1}^{N_g}\sum_{j=1}^{N_g} \frac{\mathrm{M(i,j)}}{1+(\frac{|\mathrm{i}-\mathrm{j}|^2}{N^2})}$$

12. **Autocorrelation:**

$$\sum_{i=1}^{N_g}\sum_{j=1}^{N_g} ijM(\mathrm{i},\mathrm{j})$$

13. **Cluster Prominence**

$$\sum_{i=1}^{N_g}\sum_{j=1}^{N_g}\left[i+j-u_x-u_y\right]^4 M(\mathrm{i},\mathrm{j})$$

14. **Cluster Shade**

$$\sum_{i=1}^{N_g}\sum_{j=1}^{N_g}\left[i+j-u_x-u_y\right]^3 M(\mathrm{i},\mathrm{j})$$

15. **Cluster Tendency**

$$\sum_{i=1}^{N_g}\sum_{j=1}^{N_g}\left[i+j-u_x-u_y\right]^2 M(\mathrm{i},\mathrm{j})$$

16. **Difference Entropy**

$$-\sum_{i=0}^{N_g-1} M_{x-y}(i)\log_2\left[M_{x-y}(i)\right]$$

17. **Maximum Probability:**

$$\max\left\{M(i,j)\right\}$$

18. **Sum variance**

$$\sum_{i=2}^{2N_g}(i-SE)^2 M_{x+y}(i)$$

19. **Informational measure of correlation 1 (IMC1):**

$$\frac{HXY-HXY1}{\max\left\{HX-HY\right\}}$$

20. **Informational measure of correlation 2 (IMC2):**

$$\sqrt{1-\mathrm{e}^{-2(HXY2-HXY)}}$$

21. **Inverse Difference Moment Normalized (IDMN):**

$$\sum_{i=1}^{N_g}\sum_{j=1}^{N_g}\frac{M(i,j)}{1+(\frac{\left|i-j\right|^2}{N^2})}$$

22. **Inverse Difference Normalized (IDN):**

$$\sum_{i=1}^{N_g}\sum_{j=1}^{N_g}\frac{M(i,j)}{1+(\frac{|i-j|}{N^2})}$$

Gray Level Run Length Matrix based features (GLRLM)

GLRLM based features were high-order statistical texture feature, which were defined as *P*(*i, j; θ*) to indicate the number of times j and gray level i appear consecutively in the direction *θ*.

Let:

P(*i, j; θ*) be the run-length matrix *P* for a direction *θ*, set *θ=0 and 45*

N_g be the number of discrete intensity values,

N_r be the number of different run lengths, and

N_p be the number of voxels in the ROI.

1. **Short Run Emphasis (SRE):**

$$\frac{\sum_{i=1}^{N_g}\sum_{j=1}^{N_r}[\frac{P(\mathrm{i},\mathrm{j};\theta)}{j^2}]}{\sum_{i=1}^{N_g}\sum_{j=1}^{N_r}P(\mathrm{i},\mathrm{j};\theta)}$$

2. **Long Run Emphasis (LRE):**

$$\frac{\sum_{i=1}^{N_g}\sum_{j=1}^{N_r}j^2P(\mathrm{i},\mathrm{j};\theta)}{\sum_{i=1}^{N_g}\sum_{j=1}^{N_r}P(\mathrm{i},\mathrm{j};\theta)}$$

3. **Gray-Level Nonuniformity (GLN):**

$$\frac{\sum_{i=1}^{N_g}[\sum_{j=1}^{N_r}P(\mathrm{i},\mathrm{j};\theta)]^2}{\sum_{i=1}^{N_g}\sum_{j=1}^{N_r}P(\mathrm{i},\mathrm{j};\theta)}$$

4. **Run-Length Nonuniformity (RLN):**

$$\frac{\sum_{j=1}^{N_r}[\sum_{i=1}^{N_g}P(\mathrm{i},\mathrm{j};\theta)]^2}{\sum_{i=1}^{N_g}\sum_{j=1}^{N_r}P(\mathrm{i},\mathrm{j};\theta)}$$

5. **Run Percentage (RP):**

$$\sum_{i=1}^{N_g}\sum_{j=1}^{N_r}\frac{P(\mathrm{i},\mathrm{j};\theta)}{N_p}$$

6. **Low Gray-Level Run Emphasis (LGRE):**

$$\frac{\sum_{i=1}^{N_g}\sum_{j=1}^{N_r}[\frac{P(\mathrm{i},\mathrm{j};\theta)}{i^2}]}{\sum_{i=1}^{N_g}\sum_{j=1}^{N_r}P(\mathrm{i},\mathrm{j};\theta)}$$

7. **High Gray-Level Run Emphasis (HGRE):**

$$\frac{\sum_{i=1}^{N_g}\sum_{j=1}^{N_r}i^2P(\mathrm{i},\mathrm{j};\theta)}{\sum_{i=1}^{N_g}\sum_{j=1}^{N_r}P(\mathrm{i},\mathrm{j};\theta)}$$

8. **Short Run Low Gray-Level Emphasis (SRLGE):**

$$\frac{\sum_{i=1}^{N_g}\sum_{j=1}^{N_r}\frac{P(\mathrm{i},\mathrm{j};\theta)}{i^2j^2}}{\sum_{i=1}^{N_g}\sum_{j=1}^{N_r}P(\mathrm{i},\mathrm{j};\theta)}$$

9. **Short Run High Gray-Level Emphasis (SRHGE):**

$$\frac{\sum_{i=1}^{N_g}\sum_{j=1}^{N_r}\frac{i^2P(\mathrm{i},\mathrm{j};\theta)}{j^2}}{\sum_{i=1}^{N_g}\sum_{j=1}^{N_r}P(\mathrm{i},\mathrm{j};\theta)}$$

10. **Long Run Low Gray-Level Emphasis (LRLGE):**

$$\frac{\sum_{i=1}^{N_g}\sum_{j=1}^{N_r}\frac{j^2P(\mathrm{i},\mathrm{j};\theta)}{i^2}}{\sum_{i=1}^{N_g}\sum_{j=1}^{N_r}P(\mathrm{i},\mathrm{j};\theta)}$$

11. **Long Run High Gray-Level Emphasis (LRHGE):**

$$\frac{\sum_{i=1}^{N_g}\sum_{j=1}^{N_r}i^2j^2P(\mathrm{i},\mathrm{j};\theta)}{\sum_{i=1}^{N_g}\sum_{j=1}^{N_r}P(\mathrm{i},\mathrm{j};\theta)}$$

12. **Mean:**

$$\frac{1}{2N_{\mathrm{g}}}\sum_{i=1}^{N_g}\sum_{j=1}^{N_g}[P(\mathrm{i,j})]^2$$

13. **Entropy:**

$$\sum_{i=1}^{N_g}\sum_{j=1}^{N_g}P(\mathrm{i,j})*\log_2 P(\mathrm{i,j})$$

14. **Energy:**

$$\sum_{i=1}^{N_g}\sum_{j=1}^{N_g}[P(\mathrm{i,j})]^2$$

Gray Level Size Zone Matrix based features (GLSZM)

GLSZM based features were high-order statistical texture features, which were defined as *P*(*i, j*) to indicate the areas of size j and gray level i.

Let:

P(*i, j*) be the size zone of matrix *P*,

N_g be the number of discrete intensity values,

N_r be the number of different areas sizes,

N_p be the number of voxels in the ROI.

1. **Small Zone Emphasis (SZE):**

$$\frac{\sum_{i=1}^{N_g}\sum_{j=1}^{N_r}[\frac{P(\mathrm{i,j})}{j^2}]}{\sum_{i=1}^{N_g}\sum_{j=1}^{N_r}P(\mathrm{i,j})}$$

2. **Large Zone Emphasis (LZE):**

$$\frac{\sum_{i=1}^{N_g}\sum_{j=1}^{N_r}j^2P(\mathrm{i,j})}{\sum_{i=1}^{N_g}\sum_{j=1}^{N_r}P(\mathrm{i,j})}$$

3. **Gray-Level Nonuniformity (GLN):**

$$\frac{\sum_{i=1}^{N_g}[\sum_{j=1}^{N_r}P(\mathrm{i},\mathrm{j})]^2}{\sum_{i=1}^{N_g}\sum_{j=1}^{N_r}P(\mathrm{i},\mathrm{j})}$$

4. **Zone-Size Nonuniformity (ZSN):**

$$\frac{\sum_{j=1}^{N_r}[\sum_{i=1}^{N_g}P(\mathrm{i},\mathrm{j})]^2}{\sum_{i=1}^{N_g}\sum_{j=1}^{N_r}P(\mathrm{i},\mathrm{j})}$$

5. **Zone Percentage (ZP):**

$$\sum_{i=1}^{N_g}\sum_{j=1}^{N_r}\frac{P(\mathrm{i},\mathrm{j})}{N_p}$$

6. **Low Gray-Level Zone Emphasis (LGZE):**

$$\frac{\sum_{i=1}^{N_g}\sum_{j=1}^{N_r}[\frac{P(\mathrm{i},\mathrm{j})}{i^2}]}{\sum_{i=1}^{N_g}\sum_{j=1}^{N_r}P(\mathrm{i},\mathrm{j})}$$

7. **High Gray-Level Zone Emphasis (HGZE):**

$$\frac{\sum_{i=1}^{N_g}\sum_{j=1}^{N_r}i^2P(\mathrm{i},\mathrm{j})}{\sum_{i=1}^{N_g}\sum_{j=1}^{N_r}P(\mathrm{i},\mathrm{j})}$$

8. **Small Zone Low Gray-Level Emphasis (SZLGE):**

$$\frac{\sum_{i=1}^{N_g}\sum_{j=1}^{N_r}\frac{P(\mathrm{i},\mathrm{j})}{i^2j^2}}{\sum_{i=1}^{N_g}\sum_{j=1}^{N_r}P(\mathrm{i},\mathrm{j})}$$

9. **Small Zone High Gray-Level Emphasis (SZHGE):**

$$\frac{\sum_{i=1}^{N_g}\sum_{j=1}^{N_r}\frac{i^2P(\mathrm{i},\mathrm{j})}{j^2}}{\sum_{i=1}^{N_g}\sum_{j=1}^{N_r}P(\mathrm{i},\mathrm{j})}$$

10. **Large Zone Low Gray-Level Emphasis (LZLGE):**

$$\frac{\sum_{i=1}^{N_g}\sum_{j=1}^{N_r}\frac{j^2P(\mathrm{i,j})}{i^2}}{\sum_{i=1}^{N_g}\sum_{j=1}^{N_r}P(\mathrm{i,j})}$$

11. **Large Zone High Gray-Level Emphasis (LZHGE):**

$$\frac{\sum_{i=1}^{N_g}\sum_{j=1}^{N_r}i^2j^2P(\mathrm{i,j})}{\sum_{i=1}^{N_g}\sum_{j=1}^{N_r}P(\mathrm{i,j})}$$

12. **Gray-Level Variance (GLV):**

$$\sum_{i=1}^{N_g}\sum_{j=1}^{N_r}\{\mathrm{iP(i,j)}-\frac{\sum_{i=1}^{N_g}i[\sum_{j=1}^{N_r}P(\mathrm{i,j})]}{N_gN_r}\}$$

13. **Zone-Size Variance (ZSV):**

$$\sum_{i=1}^{N_g}\sum_{j=1}^{N_r}\{\mathrm{jP(i,j)}-\frac{\sum_{i=1}^{N_g}i[\sum_{j=1}^{N_r}P(\mathrm{i,j})]}{N_gN_r}\}$$

Neighborhood Gray Tone Difference Matrix based features (NGTDM)

NGTDM based features were high-order statistical texture features, which were defined as *S(i)* to indicate the sum of the absolute value between gray intensity level i and it's neighbors' average intensity.

Let:

S(i) be the sum of absolute value between gray intensity level i and its neighbors' average intensity,

C(i) be the number of voxels with the gray intensity level I,

N_g be the number of discrete intensity values.

1. **Coarseness:**

$$\frac{1}{\varepsilon+\sum_{i=1}^{N_g}\frac{C(\mathrm{i})\mathrm{S(i)}}{\sum_{i=1}^{N_g}C(\mathrm{i})}}$$

2. **Contrast:**

$$\frac{\sum_{i=1}^{N_g}\sum_{j=1}^{N_g} C(\mathrm{i})\,\mathrm{C(j)(i-j)}^2}{(\sum_{i=1}^{N_g} C(\mathrm{i}))^2} * \sum_{i=1}^{N_g} S(\mathrm{i}) * \frac{1}{N_g(\mathrm{N}_g-1)\sum_{i=1}^{N_g} C(\mathrm{i})}$$

3. **Busyness:**

$$\frac{\sum_{i=1}^{N_g} C(\mathrm{i})\,\mathrm{S(i)}}{\sum_{i=1}^{N_g}\sum_{j=1}^{N_g} (\mathrm{iC(i)-jC(j)})}, \quad C(\mathrm{i}) \neq 0, \mathrm{C(j)} \neq 0$$

4. **Complexity:**

$$\frac{1}{(\sum_{i=1}^{N_g} C(\mathrm{i}))^2} * \sum_{i=1}^{N_g}\sum_{j=1}^{N_g} \frac{|\mathrm{i-j}|\,(\mathrm{C(i)\,S(i)+C(j)\,S(j)})}{(\mathrm{C(i)+C(j)})}, \quad C(\mathrm{i}) \neq 0, \mathrm{C(j)} \neq 0$$

5. **Strength:**

$$\frac{\sum_{i=1}^{N_g}\sum_{j=1}^{N_g} (\mathrm{C(i)-C(j)}) * (\mathrm{i-j})^2}{\sum_{i=1}^{N_g} C(\mathrm{i}) * \sum_{i=1}^{N_g} S(\mathrm{i})}, \quad C(\mathrm{i}) \neq 0, \mathrm{C(j)} \neq 0$$

(4) Wavelet features: first order statistical and texture features of a wavelet filtered image. A total of 1302 derived wavelet features were extracted for each sequence, with the Gaussian filter and a wavelet-based filter. These features were computed on the filtered images. The original image was filtered by 8 filters. For each image, the first order statistical and texture features were computed. Finally, 3906 wavelet-based features were extracted.

(II) Supporting Material: Conventional US examinations and immunohistochemical results

Breast US examinations were performed by an experienced radiologist (>10 years of experience) at two time points (before NAC, and after NAC within 3 days. We collected US images for each lesion at each scan, as follows: (1) one image was the maximum diameter of the lesion recorded; (2) the other image was acquired by rotating the probe 90° in the same position. The transverse and longitudinal images of the lesion were captured. The

measurements of the index lesions in three dimensions were obtained. The volume of the index lesion was calculated from the three greatest tumor dimensions (called the width, length, and height) using the following equation:

$$Volume = Width \times Length \times Heiht \times \frac{\pi}{6}$$

US data were obtained using a MyLab 90 ultrasound machine (Esoate, Genoa, Italy) equipped with a 4–15 MHz linear array transducer.

All patients underwent a core needle biopsy prior to NAC and immunohistochemical results, including tumor type, estrogen receptor (ER) status, progesterone receptor (PR) status, human epidermal growth factor receptor 2 (HER2), and the Ki-67 proliferation index were obtained.

(III) Supporting Material: Basic principles of deep learning and neural networks

Deep learning is based on a deep neural network architecture and learns network weights for specific tasks from big data. The neural network is composed of many different types of layers, and the layer is also the basic unit of forward calculation. In this study, the two neural networks included convolutional layers, maximum pooling layers, average pooling layers, batch normalization layers, fully connected layers, activation function layers, and a global average pooling layer. The details of these layers are explained as follows:

(1) Convolutional layer. The convolutional layer adopts some filters (kernels) with a sliding stride and kernel size to calculate the weighted sum of the intensities of each point and its surrounding points of the input matrix. Its main function is to extract the features from the input matrix. For example, assuming an input matrix $I = \begin{pmatrix} I_{11} & I_{12} & I_{13} \\ I_{21} & I_{22} & I_{23} \\ I_{31} & I_{32} & I_{33} \end{pmatrix}$ and a filter $K = \begin{pmatrix} k_{11} & k_{12} \\ k_{21} & k_{22} \end{pmatrix}$ with a kernel size of 2 × 2 and a stride of 1, the output of the convolutional layer is

$$F = conv(I, K) =$$

$$\begin{pmatrix} I_{11} * k_{11} + I_{12} * k_{12} + I_{21} * k_{21} + I_{22} * k_{22} & I_{12} * k_{11} + I_{13} * k_{12} + I_{22} * k_{21} + I_{23} * k_{22} \\ I_{21} * k_{11} + I_{22} * k_{12} + I_{31} * k_{21} + I_{32} * k_{22} & I_{22} * k_{11} + I_{23} * k_{12} + I_{32} * k_{21} + I_{33} * k_{22} \end{pmatrix}.$$

Output F is the channel of the feature map. By using multiple filters, a multi-channel feature map can be generated, which significantly improves the fitting ability of the convolutional layer. The neural network can extract more abstract features through continuous stacking of convolutional layers. In our study, the input US images can be considered a two-dimensional (2D) matrix; thus, the 2D convolutional layers were used for feature extraction.

(2) Maximum pooling layer. The maximum pooling layer is mainly used to perform feature selection and dimensionality reduction on the input feature map. Effective feature selection and dimensionality reduction can improve robustness and reduce the complexity of the deep learning model. Assuming the feature map is $F = \begin{pmatrix} 1 & 5 & 2 & 8 \\ 3 & 9 & 7 & 8 \\ 1 & 0 & 2 & 6 \\ 8 & 5 & 3 & 2 \end{pmatrix}$, whose size is 4 × 4, the pooling sliding window is 2 × 2 with a stride of 2. The pooling operation divides matrix F into four disjoint sub-matrixes of size 2 × 2, and the maximum value of each small matrix is extracted to form the result matrix $P = \begin{pmatrix} 9 & 8 \\ 8 & 6 \end{pmatrix}$. In our study, one 2D max-pooling layer was used to perform feature selection and dimensionality reduction of US images.

(3) Average pooling layer. The function of the average pooling layer is similar to that of the pooling layer. However, the calculation methods were different. For example, assuming that the feature map is also $F = \begin{pmatrix} 1 & 5 & 2 & 8 \\ 3 & 9 & 7 & 8 \\ 1 & 0 & 2 & 6 \\ 8 & 5 & 3 & 2 \end{pmatrix}$, the pooling sliding window is 2 × 2 with a stride of 2. Similar to the max-pooling layer, the average pooling operation divides the matrix F into four disjoint sub-matrixes of size 2 × 2. The average value, not the maximum value of each small matrix, will be calculated to generate the result matrix $P = \begin{pmatrix} 4.5 & 6.25 \\ 3.5 & 3.25 \end{pmatrix}$. In this study, an average pooling layer was used in each transition layer.

(4) Batch normalization layer. The batch normalization layer can normalize each channel of a batch of feature maps based on its mean and variance in the training process [1]. During the test, the statistical parameters learned from the training samples were used to normalize the test samples. Using the batch normalization layer can significantly accelerate network training and alleviate overfitting. In our model, batch normalization layers were used after or before all the convolutional layers.

(5) Fully connected (FC) layers. The fully connected layer can be described as a matrix multiplication plus one bias term, that is, $FC(x) = Wx + b$. The fully connected layer is usually used at the top of the network to provide a global receptive field for the network, in order to overcome the shortcomings of the convolutional layer that has only a limited receptive field. Through the fully connected layer, the feature map can be fused and compressed simultaneously and converted into the probability output of each category. In this study, three

fully connected layers were used for a two-category classification task.

(6) Activation-function layer. The operations in the convolutional and fully connected layers can also be expressed in the form of matrix multiplication. Matrix multiplication is a linear operation, and it is often difficult to fit complex problems using only linear operations. To strengthen the nonlinear fitting ability of the neural network, an activation function layer needs to be used. In this study, we adopted a rectified linear unit (ReLU) function layer ($ReLU(x) = max(0, x)$) after most convolutional layers and fully connected layers in our networks.

(7) Global average pooling (GAP) layer. The function of the GAP layer is to calculate the average intensity of all elements in each channel of a feature map, and finally output a feature vector whose length is the same as the number of channels. The use of a GAP layer can significantly reduce the number of weights in the network and effectively alleviate overfitting. In this study, we used the GAP layer between the last convolutional layer and the first fully connected layer.

(IV) Supporting Material: Statistic metric

In our study, the following six quantitative indicators were used to evaluate the performance of our models: the receiver operating characteristic curve (AUC), accuracy, sensitivity, specificity, and negative predictive value (NPV). The formulas used to calculate these indicators are as follows:

1. AUC:

$$AUC = \frac{\sum_{ins_i \in positiveclass} rank_{ins_i} - \frac{M \times (M+1)}{2}}{M \times N},$$

where M and N are the number of positive and negative samples, respectively. $rank_{ins_i}$ is the serial number of sample i.

2. Accuracy:

$$accuracy = \frac{TP + TN}{TP + TN + FP + FN}$$

3. Sensitivity:

$$sensitivity = \frac{TP}{TP + FN}$$

4. Specificity:

$$specificity = \frac{TN}{TN + FP}$$

5. NPV:

$$NPV = \frac{TN}{TN + FN}$$

where TP, TN, FP, and FN are the numbers of true positive, true negative, false positive, and false negative samples, respectively.

Figure S2. Radiomics features selection using the least absolute shrinkage and selection operator (LASSO) regression model. The five-fold cross validation method was used in LASSO to screen the feature sets with the best performance and the 1-standard error criteria and the value λ was chosen (A). LASSO coefficient profiles for selected features at optimal λ values, which result in non-zero coefficients for the selected features (B). (2-column fitting image)

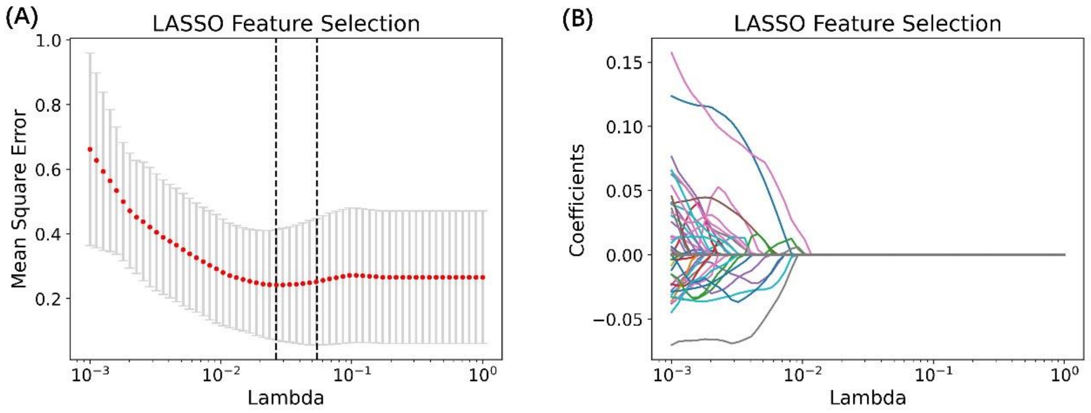

Supplement: Supplementary file 1 — Supplementary Materials [file 330_2024_10786_MOESM1_ESM.pdf]
